# Case Report: Avermectin poisoning-associated hemolytic uremic syndrome

**DOI:** 10.3389/fimmu.2026.1753201

**Published:** 2026-04-23

**Authors:** Jianping Xiao, Rui Shi, Qi Cao, Deguang Wang, Ruifeng Wang

**Affiliations:** 1 Department of Nephrology, The Second Affiliated Hospital of Anhui Medical University, Hefei, Anhui, China; 2 Centre for Transplant and Renal Research, Westmead Institute for Medical Research, The University of Sydney, Sydney, NSW, Australia

**Keywords:** acute kidney injury, avermectin poisoning, complement activation, eculizumab, thrombotic microangiopathy

## Abstract

Avermectin, a commonly used agricultural pesticide and antiparasitic agent, typically causes acute human toxicity characterized by central nervous system depression and gastrointestinal disturbances. However, its association with thrombotic microangiopathy has not been previously reported. We describe a 60-year-old woman who developed anuria after ingesting 300 mL (15 g) of avermectin. Laboratory investigations demonstrated thrombocytopenia, microangiopathic hemolytic anemia, and acute kidney injury. Renal biopsy revealed thrombotic microangiopathy-like lesions. Thrombotic thrombocytopenic purpura and Shiga toxin-producing *Escherichia coli*-associated hemolytic uremic syndrome were excluded. Markedly elevated plasma levels of the terminal complement complex (C5b-9) confirmed complement activation, supporting the diagnosis of complement-mediated thrombotic microangiopathy. The patient subsequently received complement inhibition therapy with eculizumab during the disease course. Hematologic abnormalities resolved and renal function gradually recovered, allowing discontinuation of dialysis; however, a definitive causal relationship between eculizumab administration and clinical recovery cannot be established. This case suggests that avermectin poisoning may act as a potential trigger of complement-mediated TMA and highlights the importance of considering this in patients with severe pesticide intoxication complicated by unexplained TMA features.

## Introduction

Avermectin, a γ-aminobutyric acid (GABA) agonist derived from *Streptomyces avermitilis*, exerts potent pesticidal and antiparasitic effects by disrupting neural signal transmission in invertebrates (1, 2). Because of its broad-spectrum activity and high efficacy, avermectin and its derivatives have been widely used in agriculture and veterinary medicine since the 1990s (3, 4). Despite their widespread application in veterinary medicine, avermectins have been shown to exert host toxicity, including neurotoxic, hepatotoxic, nephrotoxic, and reproductive effects, through interactions with γ-aminobutyric acid and glutamate-gated chloride channels (5). In human, however, the toxic profile of avermectins remains incompletely characterized. Acute poisoning, though rare, is dominated by neurotoxicity, including coma, ataxia, and hypotension, sometimes complicated by aspiration pneumonia (6, 7). To date, thrombotic microangiopathy (TMA) has not been associated with avermectin poisoning.

Atypical hemolytic uremic syndrome (aHUS) is a rare complement-mediated TMA characterized by microangiopathic hemolytic anemia, thrombocytopenia, and ischemic organ injury (8). In the traditional classification, aHUS is distinguished from secondary TMAs by its primary association with dysregulated activation of the alternative complement pathway, often due to pathogenic variants in complement regulatory genes or acquired autoantibodies such as anti-factor H (9). In contrast, secondary TMAs are triggered by external factors including infections, malignant hypertension, pregnancy, transplantation, and certain medications (10–12).

Although complement blockade with agents such as eculizumab is clearly indicated as first-line therapy in primary complement-mediated aHUS, its role in secondary TMAs remains uncertain and is not universally recommended (13). Nevertheless, increasing evidence suggests that complement overactivation may contribute to the pathogenesis of a subset of secondary TMAs, leading some experts to propose a broader concept of complement-mediated TMA (14). However, the indication for complement inhibition in these cases remains controversial and should be individualized based on clinical context and evidence of complement activation.

Here, we report the first case of avermectin poisoning-associated TMA with evidence of complement activation. Although the causal role of complement inhibition cannot be conclusively determined, this case highlights the potential for avermectin to trigger complement dysregulation and emphasizes the need for careful evaluation of complement activity in severe intoxication-related TMA.

## Case report

A 60-year-old woman was found unconscious on July 6, 2025 (Day 0) following intentional ingestion of approximately 300 mL (15 g) of avermectin in a suicide attempt. She was discovered by family members about 2 hours after ingestion and admitted to the intensive care unit, where gastric lavage, intravenous fluid resuscitation, and endotracheal intubation were performed. No co-ingestion of other substances was reported. Shortly after admission, she developed anuria (urine output <100 mL/24h) with progressive elevation of serum creatinine (Scr), peaking at 256 μmol/L. Despite 4 days of hemoperfusion for toxin clearance and continuous renal replacement therapy (CRRT) for acute kidney injury (AKI), there was no improvement in consciousness or renal function. On July 10 (Day 4), she was transferred to our hospital, where hemoperfusion and CRRT were continued. She gradually regained consciousness, but remained anuria and dialysis-dependent. On July 19 (Day 13), she was admitted to the Department of Nephrology for further evaluation and management.

The patient had no recent history of infection, diarrhea, fever, excessive sweating, decreased appetite, or use of special medications in the days preceding the ingestion. There was also no history of hypertension, diabetes mellitus, chronic kidney disease, or other chronic illnesses. A routine health examination performed one week prior to ingestion revealed normal blood counts, urinalysis, and liver and kidney function, indicating that she was in good health before the event.

On admission to our hospital (Day 4), vital signs were as follows: temperature 36.0 °C, pulse 84 beats/min, respiratory rate 18 breaths/min, and blood pressure 77/47 mmHg; body mass index was14.5 kg/m^2^. Physical examination revealed deep coma with anisocoria (pupil sizes 1.5 and 2.5 mm). Coarse breath sounds with scattered rales were heard bilaterally, and the abdomen was soft without organomegaly. Laboratory evaluation at admission revealed anemia, severe thrombocytopenia, elevated schistocyte, and markedly increased lactate dehydrogenase (LDH) (**Table 1**). Blood and urine avermectin concentrations are summarized in **Table 2**.

**Table 1:** Laboratory test results.

Date	Laboratory test	Result (normal range)
July 10	White blood cells (×1,000/mm^3^)	10.18 (3.5-9.5)
	Hemoglobin (g/L)	87 (115–150)
	Hematocrit (percent)	0.25 (0.35-0.45)
	Platelet count (×1,000/mm^3^)	30 (125–350)
	Urea (mmol/L)	14.81 (3.1-8.8)
	Creatinine (umol/L)	243 (41–81)
	Aspartate transaminase (U/L)	221 (7–40)
	Alanine transaminase (U/L)	184 (13–35)
	Lactate dehydrogenase (U/L)	3009 (120–250)
	Creatine phosphokinase (U/L)	112 (40–200)
	Alkaline phosphatase (IU/L)	80 (50–135)
	Total bilirubin (umol/L)	45.2 (0–21)
	Direct bilirubin (umol/L)	12.5 (0-6.8)
July 21	C3 (g/L)	0.52
	C4 (g/L)	0.1
July 22	urinary protein	2+
	urinary red blood cells	88/uL
	urine specific gravity	1.006
July 27	schistocyte percentage	3%
	ADAMTS13 activity %	125.57 (42.16-126.37)
	ADAMTS13 activity-inhibiting antibody	Negative
	C5b-9 (ng/mL)	320 (75–219)

**Table 2:** The Avermectin levels in different samples.

Sample type	Avermectin concentration (ug/mL)	
	July 8 (Day 2)	July 11 (Day 5)
Blood	0.025	0.007
Urine	0.001	0.002
Stomach	/	0.005

After transfer to our nephrology department on July 19 (Day 13), blood pressure had stabilized at 130/56 mmHg, shock had been corrected, and consciousness had recovered. However, the patient remained anuric and dialysis-dependent with no signs of renal recovery. Urinalysis performed on July 22 (Day 16) demonstrated low specific gravity (1.006) and proteinuria (2+), suggestive of combined tubular and glomerular involvement. However, quantitative proteinuria assessment was not feasible due to severe oliguria at that time. On July 27 (Day 21), peripheral blood smear revealed a marked increase in schistocytes. Although platelet count and LDH had improved compared with admission values, LDH remained elevated above 1.5× the upper limit of normal. Persistent anuria with ongoing tubular and glomerular abnormalities raised high suspicion for thrombotic microangiopathy (TMA).

To further clarify the etiology and evaluate for atypical hemolytic uremic syndrome (aHUS), additional investigations were performed on July 27 (Day 21). ADAMTS13 activity and antibody levels were within normal limits, and Stool culture was negative for *Shiga* toxin-producing *Escherichia coli*. Complement testing demonstrated markedly elevated terminal complement complex (C5b-9) levels, indicating ongoing complement activation.

**Figure 1:**
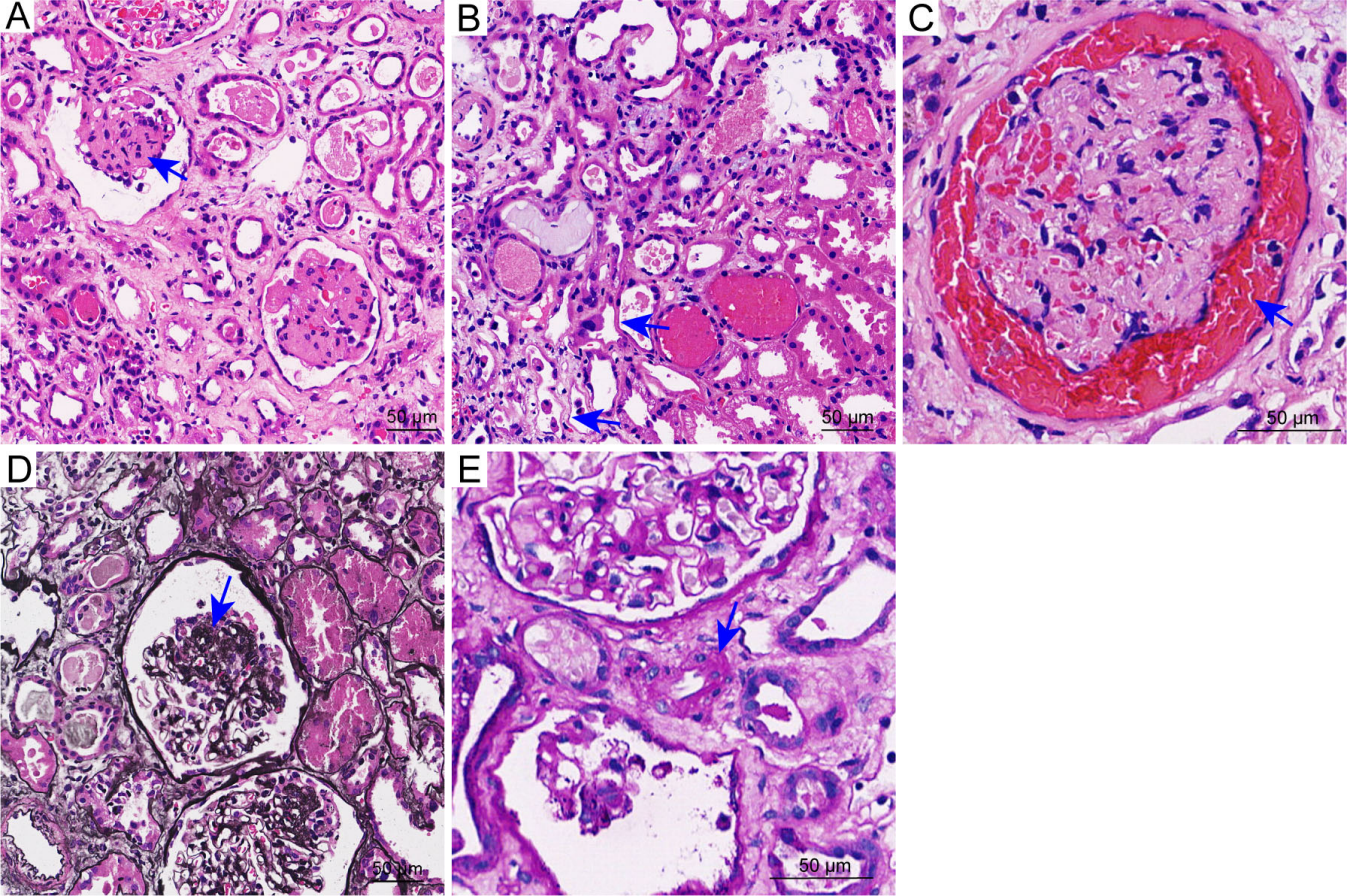
Representative renal biopsy images. **(A)** Global collapse and occlusion of glomerular capillary loops with ischemic sclerosis (blue arrow) (H&E stain, original magnification ×200). **(B)** Tubular epithelial injury, including epithelial cell swelling, cytoplasmic eosinophilia, loss of brush borders, and focal epithelial detachment (blue arrow). The surrounding interstitium exhibits mild to moderate edema with inflammatory cell infiltration (H&E stain, original magnification ×200). **(C)** Red blood cell stasis within Bowman’s space (blue arrow) (H&E stain, original magnification ×200). **(D)** Segmental collapse, occlusion, and ischemic changes of the glomerular capillary loops, with podocyte hyperplasia along the peripheral loops (blue arrow) (Jones silver stain, original magnification ×200). **(E)** A small interlobular artery showing mild wall thickening (blue arrow) (PAS stain, original magnification ×400).

Given persistent anuria and dialysis dependence, a percutaneous renal biopsy was performed on July 23, 2025 (Day 17). Histopathology revealed TMA-like lesions predominantly in glomeruli, including capillary loop occlusion, ischemic alterations, and red blood cell stasis in the Bowman’s space. Tubules and interstitium showed severe acute injury with vacuolar and granular degeneration of tubular epithelial cells, intraluminal erythrocyte and protein casts, cellular debris, focal epithelial exfoliation, and denuded basement membranes. In the renal interstitium, only a few small arteries showed mild wall thickening, while no definite arteriolar thrombi, fibrinoid necrosis, or significant vascular inflammation were observed (**Figure 1**). Therefore, vascular involvement was primarily glomerular. Genetic testing identified a variant of uncertain significance in the *ITGA2B* gene (NM_000419.5:c.2602-3C>G).

The patient exhibited the classical triad of TMA, including microangiopathic hemolytic anemia (anemia, schistocytes, elevated LDH), severe thrombocytopenia, and acute kidney injury with anuria. TTP was excluded by normal ADAMTS13 activity and absence of anti-ADAMTS13 antibodies. STEC-HUS was excluded by negative stool culture for Shiga toxin–producing *Escherichia coli*. Renal biopsy confirmed TMA-like lesions with glomerular occlusion, red blood cell stasis in Bowman’s space, and severe tubulointerstitial injury. Markedly elevated plasma terminal complement complex (C5b-9) supported ongoing complement activation. In the absence of other identifiable causes and in the context of avermectin poisoning, a diagnosis of complement-associated TMA was considered.

Eculizumab therapy was initiated after consideration of complement-associated TMA, based on the following rationale: despite correction of shock, recovery of consciousness, and toxin clearance, the patient remained anuric with persistent TMA features (LDH >1.5× the upper limit of normal, ongoing tubular and glomerular injury on urinalysis), and renal biopsy showed TMA-like changes. Complement testing (C5b-9 and C3) indicated sustained activation, supporting the use of terminal complement inhibition.

**Figure 2:**
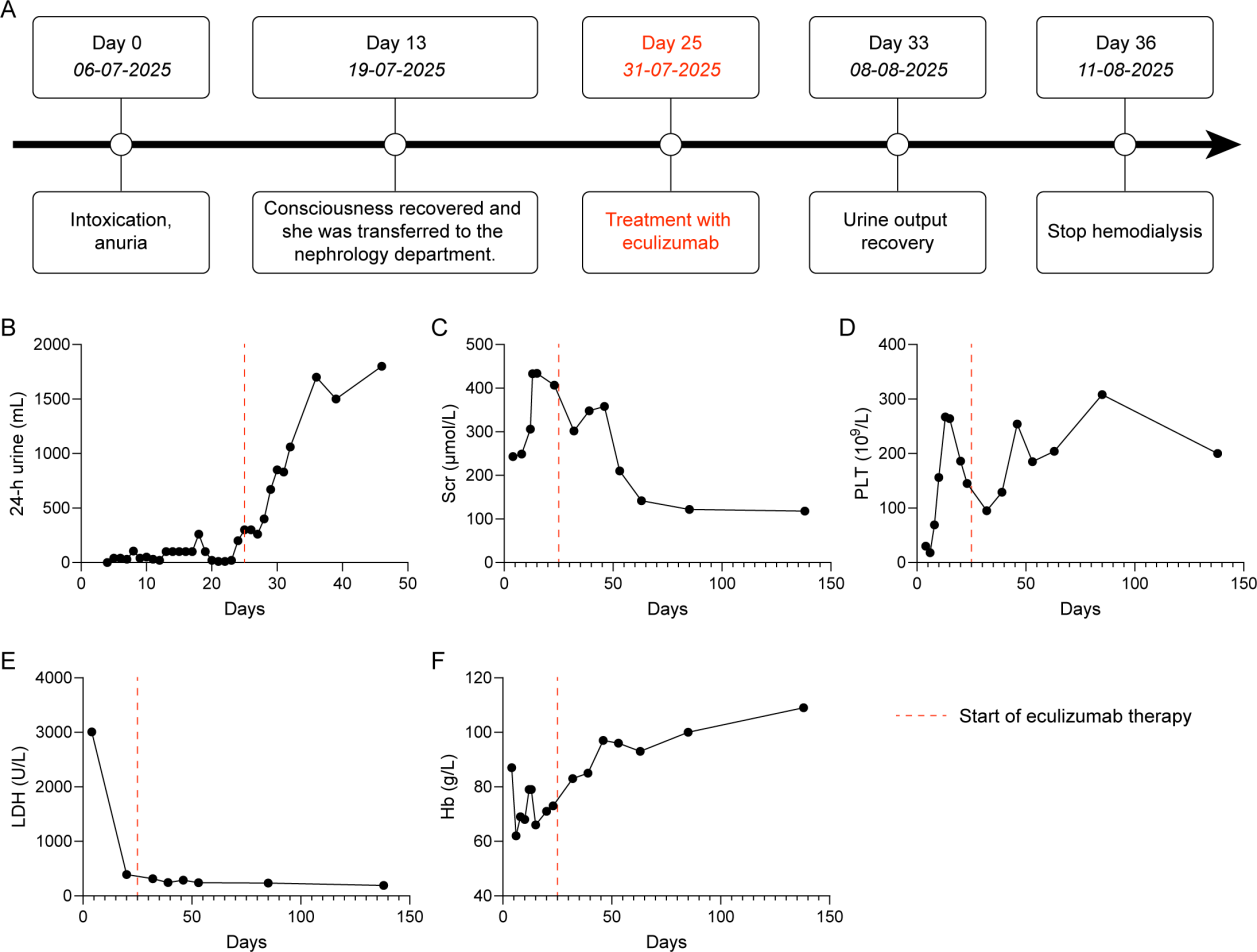
Treatment course and trends in laboratory parameters. **(A)** Timeline of disease onset, diagnosis, treatment, and clinical outcomes. **(B-F)** Dynamic changes in diagnostic parameters over the course of treatment. Scr, serum creatinine; PLT, platelets; LDH, lactate dehydrogenase; Hb, Hemoglobin.

Following eculizumab treatment, urine output gradually increased, reaching 1700 mL/24h by August 11 (Day 67), allowing discontinuation of dialysis. Serum creatinine level decreased from 433 μmol/L (July 19, Day 13) to 140 μmol/L (September 9, Day 95). Platelet counts normalized to 254×10^9^/L by August 21 (Day 77), LDH returned to normal (289 U/L), and hemoglobin improved to 105 g/L (**Figure 2**). Eculizumab was discontinued three months after renal function recovery. The dosing regimen and administration frequency of eculizumab are summarized in **Table 3**.

**Table 3:** Eculizumab dosing regimen and administration frequency during treatment.

Date	Dose (mg)
July 31 (Day 25)	900
August 7 (Day 32)	900
August 14 (Day 39)	900
August 21 (Day 46)	900
August 28 (Day 53)	1200
September 29 (Day 85)	1200
November 21 (Day 138)	1200

At follow-up on March 30, 2026, the patient’s renal function remained stable without evidence of relapse, supporting the possibility that short-term complement inhibition may be sufficient in toxin-triggered TMA, although longer-term observation is warranted.

## Discussion

We report the first case of TMA following avermectin poisoning, in which a diagnosis of complement-mediated TMA was considered. This case raises the possibility of an association between avermectin poisoning and complement-mediated TMA, which not only fills the gap in research on the mechanism of avermectin-induced nephrotoxicity but also provides crucial references for the clinical diagnosis and management of pesticide poisoning complicated with TMA.

Avermectin is a widely used antiparasitic and agricultural pesticide whose toxicological profile in humans remains incompletely defined. Previous studies of acute avermectin intoxication have primarily described central nervous system depression and respiratory complications (6). To date, no direct association between avermectin exposure and complement-mediated TMA has been established. In this case, after avermectin poisoning, the patient developed the classic TMA triad: microangiopathic hemolytic anemia, thrombocytopenia, and acute kidney injury, with renal biopsy confirming TMA-like lesions and laboratory studies revealing elevated C5b-9, consistent with complement activation.

Mechanistically, although avermectin exerts its pesticidal effect through γ-aminobutyric acid agonism and neural signaling disruption, recent studies suggest that it may also damage vascular endothelial cells through mitochondria dysfunction and perturbations of the VEGF/Notch pathway (3). Vascular endothelial injury is a well-recognized trigger for dysregulated activation of the alternative complement pathway in aHUS, resulting in uncontrolled endothelial C5b-9 deposition, endothelial swelling and dysfunction, platelet activation, and formation of microthrombi, key pathological features of aHUS (15). Thus, this case provides novel insight into the nephrotoxic potential of avermectin beyond its neurotoxicity, supporting an endothelial injury-complement activation axis as a plausible mechanism.

Genetic testing of the patient revealed a variant of uncertain significance in *ITGA2B* gene (NM_000419.5:c.2602-3C>G). *ITGA2B* gene encodes the α-subunit of platelet glycoprotein IIb (GPIIb), which combines with GPIIIa to form the GPIIb/IIIa integrin receptor, a central mediator of platelet adhesion and aggregation (16). Pathogenic *ITGA2B* variants are classically associated with Glanzmann thrombasthenia, a bleeding disorder characterized by defective platelet aggregation (17, 18). Although the clinical relevance of the identified variant remains uncertain, it may have contributed as a permissive factor by subtly altering platelet responsiveness to complement activation. This could enhance platelet aggregation and microthrombus formation in the context of complement-mediated endothelial injury. Nevertheless, the dominant pathogenic driver in this case appears to be avermectin-induced complement dysregulation, with the *ITGA2B* variant possibly lowering the threshold for clinical manifestation of complement-mediated TMA.

The differential diagnosis of TMA is complex and requires careful distinction between primary aHUS and secondary TMAs triggered by identifiable conditions. Primary aHUS is driven by dysregulated activation of the alternative complement pathway, typically associated with pathogenic complement gene variants or acquired complement abnormalities, and complement inhibition is an established therapeutic strategy in this setting. In contrast, secondary TMAs arise in association with external or systemic triggers, including infections, malignant hypertension, pregnancy, autoimmune diseases, transplantation, and drug or toxin exposure (19, 20). In these conditions, endothelial injury related to the triggering factor is considered the primary pathogenic event, whereas complement activation may occur as a downstream or amplifying mechanism rather than the initiating driver (21, 22). In the present case, severe hypotension at presentation likely contributed to an initial ischemic insult, and short-term vasopressor use may have exacerbated microvascular hypoperfusion. However, the persistence of TMA features beyond hemodynamic stabilization, together with hematologic and histopathological findings, suggests that these factors alone do not account for the observed disease course. Importantly, many cases of secondary TMAs improve with removal of the trigger and supportive management alone.

Drug- and toxin-associated TMA represent a heterogeneous subgroup within secondary TMA (23, 24). In most cases, withdrawal of the offending agent and supportive care lead to clinical improvement. However, persistent or severe disease despite effective elimination of the trigger has raised the possibility that complement overactivation may contribute to disease propagation in selected cases. In the present case, despite timely clearance of avermectin, as documented by declining blood concentrations (**Table 2**), and the application of standard supportive therapies including renal replacement therapy, the patient remained anuric with ongoing hematologic features of TMA for a prolonged period. This clinical course prompted consideration of additional pathogenic mechanisms beyond direct toxic injury. Persistence of TMA despite removal of the trigger may prompt consideration of underlying complement susceptibility; nevertheless, such cases are best regarded as secondary TMAs with complement involvement. Together, these observations underscore the heterogeneity of secondary TMAs and the need for cautious interpretation of complement activation findings in clinical practice.

Eculizumab, a terminal complement inhibitor targeting C5, has revolutionized the management of primary aHUS by preventing irreversible organ damage and progression to end-stage renal disease (25–27). In this case, eculizumab was initiated during the clinical course after diagnosis of complement-associated TMA was considered. The decision was based on multiple converging lines of evidence suggesting ongoing complement-mediated microvascular injury despite initial supportive management. Although systemic conditions such as shock had been corrected, consciousness had recovered, and toxin exposure had been discontinued, the patient remained anuric with persistent laboratory and urinary abnormalities indicative of active TMA, including elevated lactate dehydrogenase (>1.5× upper limit of normal) and evidence of ongoing tubular and glomerular injury on urinalysis. In addition, renal biopsy demonstrated TMA-like microangiopathic changes, and complement testing revealed sustained activation with abnormal C3 deposition and increased terminal complement activity (C5b-9), collectively supporting ongoing complement pathway involvement and providing a rationale for terminal complement inhibition.

Prior to initiation of eculizumab, the patient had persistent oliguria/anuria, and therefore only routine urinalysis could be performed on July 22. Quantitative assessment of proteinuria and characterization of protein subtype were not feasible due to insufficient urine output. Despite this limitation, urinalysis demonstrated proteinuria (2+) with low urine specific gravity (1.006), suggesting significant renal parenchymal injury involving both tubular dysfunction and possible glomerular involvement. This interpretation is further supported by subsequent renal biopsy findings, which demonstrated combined tubular injury and glomerular TMA-like lesions, thereby providing histopathological confirmation of multi-compartment renal involvement.

Hematologic parameters and renal function subsequently improved following eculizumab administration. However, given that partial recovery had already commenced prior to complement inhibition, a definitive therapeutic effect of eculizumab cannot be determined. Spontaneous recovery and/or the cumulative effects of supportive care cannot be excluded as alternative explanations for the observed clinical improvement. The clinical course underscores the importance of considering complement-associated TMA in patients with intoxication-associated AKI and TMA features, particularly when conventional therapies fail. Although eculizumab is an established therapy for primary aHUS, its role in secondary or trigger-associated TMAs, including toxin-related cases, remains uncertain. In addition, the lack of serial post-treatment complement measurements, including sC5b-9, precludes direct pharmacodynamic assessment of complement inhibition. Consequently, the effectiveness of eculizumab was inferred from the temporal association with clinical and hematologic recovery rather than confirmed by laboratory markers. Therefore, the present case does not allow conclusions regarding treatment efficacy but rather highlights the diagnostic challenges and therapeutic uncertainty in intoxication-associated TMA.

In conclusion, this case suggests that avermectin poisoning may be a potential trigger for complement-associated TMA. The findings underscore the importance of considering complement-associated TMA in patients with severe intoxication complicated by unexplained TMA. However, the therapeutic role of complement inhibition in this setting remains uncertain, and further clinical experience is required to clarify the relationship between toxin exposure, complement activation, and treatment response.

## Data Availability

The original contributions presented in the study are included in the article/supplementary material. Further inquiries can be directed to the corresponding author.
